# Relationship between the Duration of the Second Stage of Labour and Neonatal Morbidity

**DOI:** 10.3390/jcm8030376

**Published:** 2019-03-18

**Authors:** Nuria Infante-Torres, Milagros Molina-Alarcón, Juan Gómez-Salgado, Julián Rodríguez-Almagro, Ana Rubio-Álvarez, Antonio Hernández-Martínez

**Affiliations:** 1 Gutiérrez Ortega Hospital, Valdepeñas, Ciudad Real 13300, Spain; nuria.i.t@hotmail.com; 2 Department of Nursing of University of Castilla la Mancha, Albacete 02071, Spain; milagros.molina@uclm.es; 3 Department of Nursing, Faculty of Nursing, University of Huelva, Huelva 21071, Spain; jgsalgad@gmail.com; 4 Espíritu Santo University, Guayaquil 092301, Ecuador; 5 Department of Nursing of University of Castilla la Mancha, Ciudad Real 13071, Spain; 6 Obstetrics Service, Torrejón of Ardoz Hospital, Madrid 28850, Spain; a.rubio.alvarez@hotmail.es; 7 Mancha-Centro Hospital, Alcázar de San Juan, Ciudad Real 13600, Spain; antomatron@gmail.com

**Keywords:** labour: second stage, newborn care, neonatal morbidity, quantitative research

## Abstract

(1) Background: To assess the relationship between the duration of the second stage of labour and the neonatal morbidity risk; (2) Methods: An observational, analytical, retrospective cohort study was performed at the “Mancha-Centro” Hospital (Spain) during the 2013–2016 period. Data were collected from 3863 women who gave a vaginal birth. The studied neonatal morbidity variables were umbilical cord arterial pH, 5-min Apgar score, need for advanced neonatal resuscitation, and a composite neonatal morbidity variable on which the multivariate analysis was done. A univariate analysis was used for the potential risk factors and a multivariate analysis with binary logistic regression to control for possible confounding factors; (3) Results: The univariate analysis showed a statistically significant relationship between the duration of the second stage of labour and a high risk of advanced neonatal resuscitation and composite neonatal morbidity in multiparous women. However, after performing the multivariate analysis for the variable “composite neonatal morbidity”, we observed no relationship with the duration of the second stage of labour in either nulliparous or multiparous women; (4) Conclusions: The duration of the second stage of labour was not related to an increased risk of neonatal morbidity in our study population.

## 1. Introduction

The second stage of labour begins when the cervix becomes fully dilated and ends with the delivery of the neonate [1]. Optimally managing the second stage of labour is a constant challenge for professionals in the clinical practice [2]. The main objective of this stage of labour is to lower caesarean section rates, increase the possibility of vaginal birth and, in turn, avoid adverse effects for both the mother and the newborn [2,3].

The second stage of labour is characterised by an increasing number and intensity of uterine contractions with respect to the first stage of labour, as well as an increase in maternal bearing down efforts, which leads to maternal fatigue and high foetal lactic acid levels [4]. If the foetus’ head lowers, it can compress the umbilical cord, which reduces cerebral perfusion [4]. Therefore, the presence or combination of these factors means that the second stage of labour is a very stressful period for the foetus.

For this reason, the duration of the second stage of labour is particularly interesting as its optimum duration is a matter of constant debate [5]. Nowadays, it is uncertain if a time point exists during the second stage of labour at which the risk of neonatal morbidity increases, leading to the need for professionals to intervene so as to prevent adverse events [6,7].

Until 2012, a prolonged second stage of labour had been defined as a period of time that lasted beyond 2 h with epidural analgesia (EA) or 1 h without EA for multiparous women. As for nulliparous women, a second prolonged stage is defined as a period of time longer than 3 h with EA or longer than 2 h without EA [8]. More recently, though, the National Institute for Health and Care Excellence (NICE) and the American College of Obstetricians and Gynecologists (ACOG) have included longer durations in some cases, although the management of the situation should be individualised according to how the delivery progresses, the use of EA, or foetal malposition [1,9]. For example, the Eunice Kennedy Shriver National Institute of Child Health and Human Development document suggested allowing one additional hour for the use of epidural analgesia. Thus, at least 3 h in multiparous women and 4 h in nulliparous women would be considered to diagnose a prolonged second stage of labour [1].

Accordingly, many authors have studied the duration of the second stage of labour and have based their definition on different guidelines and its relationship with neonatal morbidity. Some of these studies have found no association between the prolonged second stage and adverse neonatal outcomes [7,10,11,12,13]. However, other studies have observed that prolonging the second stage of labour increases the risk of being admitted to a Neonatal Intensive Care Unit (NICU) or a low 5-min Apgar score [2,3,14,15,16,17,18,19].

Reducing the duration of the second stage of labour by obstetric interventionism is not a complication-free solution as, paradoxically, some of these interventions include immediate pushing (initiated as soon as complete dilation is identified) [20], fundal pressure [21], or instrumental birth [22], actions that may increase the risk of neonatal morbidity.

Therefore, given today’s high level of uncertainty and the important implications that the time limitation of the second stage of labour has for the clinical practice, we propose conducting this study to assess the relationship between the duration of the second stage of labour and the neonatal morbidity risk.

## 2. Materials and Methods

### 2.1. Design and Participants

An observational, analytical, retrospective cohort study was done on a sample of 3863 women who had given birth vaginally at the “Mancha-Centro Hospital” in Spain, during the 2013–2016 period. This is a Level II centre attending nearly 1300 births a year, and the hospital’s global rate of caesareans amounts to 25.3%. The vaginal delivery rate after a previous caesarean section was 38%. The centre has a NICU and, in labours without complications, the main responsible professionals are the midwives, while in the case of labours with complications or instrumental labours, this role is played by obstetricians. For the limitation of the duration of the first and second stages of labour, the recommendations by the Spanish GPC are followed as a guideline. Regarding induced labours, induction failure is diagnosed if, after 12 h of labour with regular uterine dynamics, the active phase of labour was not reached. In this active phase, non-progressive labour is considered when there are no changes regarding cervical dilatation during 4 h with regular uterine dynamics.

A prolonged second stage of labour had been defined as a period of time that lasted beyond 3 h with EA or 2 h without EA for multiparous women. As for nulliparous women, a second prolonged stage is defined as a period of time longer than 4 h with EA or longer than 3 h without EA.

The inclusion criterion was vaginal births, including cephalic presentation and singleton births. Any births with a gestational age <35 weeks and with antepartum foetal death were excluded.

### 2.2. Sources of Information

For data collection, the women’s medical records and those of the study neonates were used.

The following variables were considered. The “primary outcome” variable was neonatal morbidity, which was divided into four types—pH <7.10, 5-min Apgar scores <7, advanced neonatal resuscitation (type III: Oxygen therapy with positive intermittent pressure, IV: Endotracheal intubation, V: Cardiac massage and/or using drugs), and composite neonatal morbidity (the combination of any of these three types). Umbilical cord arterial pH is one of the best predictors of perinatal adverse outcomes [23,24], and the threshold pH for adverse neurological outcomes is 7.10 [25]. The Apgar score describes the condition of the newborn infant immediately after birth and, when properly applied, it is a tool for standardised assessment. If the Apgar score at 5 min is greater than or equal to 7, it is unlikely that peripartum hypoxia–ischemia causes neonatal encephalopathy [26]. The main independent variable was the duration of the second stage of labour (up to 1 h/1–2 h/2–3 h/≥3 h).

The secondary independent variables taken into account as potentially confounding factors were neonatal birth weight (≤2500 g/2500–3999 g/≥4000 g), labour induction (yes/no), gestational age (<37 weeks/37–41 weeks/>41 weeks), duration of the first stage of labour (up to 3 h/>3–6 h/>6–9 h/>9 h), epidural analgesia (yes/no), type of birth (normal/instrumental), maternal age (≤35 years/>35 years), and previous caesarean birth in multiparous women (yes/no).

#### Statistical Analysis Used

A univariate analysis of the potentially predictive factors was carried out by using the Chi-squared tests to calculate the categorical variables, stratified for nulliparous and multiparous women (Table 1 and Table 2). Then, a multivariate analysis was performed by binary logistic regression, where all the variables considered potential risk factors of neonatal morbidity for both nulliparous and multiparous women were used. The statistical analysis was performed in a stratified way for nulliparous and multiparous, since the parity variable is considered an effect modifying factor.

The “primary outcome” variable was a composite of neonatal morbidity (CNM yes/no) (Table 3). The results were analysed using the SPSS 24.0 statistical package (SPSS Inc., Chicago, IL, USA).

### 2.3. Ethical–Legal Considerations

This study was approved by the centre’s Ethics in Clinical Research Committee, which guaranteed the confidentiality of the medical records and the information they contained at all times.

## 3. Results

**Figure 1:**
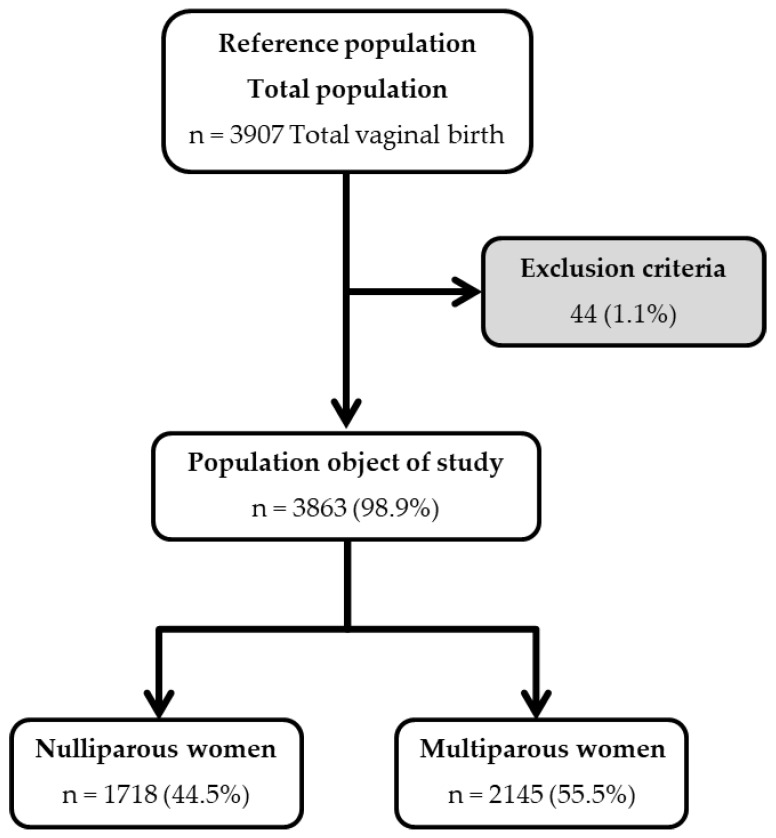
Selection of the study subjects.

The study was initiated with a reference population of 3907 women, and after applying the exclusion criteria, the study population consisted of 3863 women. Of these, 1718 (44.5%) women were nulliparous and 2145 (55.5%) were multiparous (Figure 1). The information was complete for all the studied variables except for umbilical cord arterial pH, as this type of data was not recorded for 164 nulliparous and 233 multiparous women. 

Once the univariate analysis was performed considering the potential risk factors, and the variables were used as the neonatal morbidity criteria according to parity, we observed a statistically significant relationship in both the nulliparous and multiparous women (*p* ≤ 0.05) between the duration of the first stage of labour and the degree of advanced neonatal resuscitation, as well as with composite morbidity. In addition, a statistically significant association was found between the type of birth and pH <7.10, advanced neonatal resuscitation, and composite morbidity (Table 1 and Table 2).

Moreover, a statistically significant relationship was found only in nulliparous women for labour induction with advanced neonatal resuscitation (Table 1).

Regarding multiparous women, a statistically significant relationship was found for labour induction with pH <7.10 and composite neonatal morbidity; for the duration of the second stage of labour with advanced neonatal resuscitation and composite neonatal morbidity; for epidural analgesia with both advanced neonatal resuscitation and composite neonatal morbidity; for maternal age with pH <7.10, advanced neonatal resuscitation, and composite neonatal morbidity; and for having had a previous caesarean birth in multiparous women with pH <7.10 and composite morbidity (Table 2). Neither the neonatal birth weight nor gestational age was statistically related to any of the neonatal morbidity variables studied in multiparous women.

None of the studied risk factors was statistically related to a 5-min Apgar score <7 in both nulliparous and multiparous women.

Then, a multivariate analysis was performed for the variable “composite morbidity” in both nulliparous and multiparous women (Table 3). In both groups, a statistically significant relationship was found with the type of birth, and in such a way that instrumental birth increased the risk of composite neonatal morbidity in nulliparous (odds ratio (OR), 2.33; 95% confidence interval (CI), 1.38–3.96), and multiparous women (OR, 5.37; 95% CI, 2.73–10.56). A statistically significant relationship was also found regarding multiparous women between neonatal birth weight and maternal age, where a low birth weight implied a higher risk of composite neonatal morbidity than a normal birth weight (OR, 2.78; 95% CI, 1.02–7.58). Maternal age >35 years also increased the risk of composite morbidity (OR, 1.97; 95% CI, 1.22–3.17). No statistically significant relationship was found between composite neonatal morbidity and labour induction, gestational age, duration of the first stage of labour, duration of the second stage of labour, or epidural analgesia.

## 4. Discussion

The objective of this study was to assess the relationship between the duration of the second stage of labour and the different neonatal morbidity criteria. No relationship between the duration of the second stage of labour and an increased risk of neonatal morbidity was observed in the study population.

Several authors have studied the influence of the duration of the second stage of labour on maternal and neonatal outcomes [2,3,7,10,11,12,13,14,15,16,17,18,19,27]. Most studies have found a relationship between the duration of the second stage of labour and an increased risk of neonatal morbidity [2,3,14,15,16,17,18,19]. Some particularly relevant works are those by Sandström et al. [2], Grobman et al. [19], Laughon et al. [18], and Zipori et al. [27], given their large sample sizes and their recent publication. However, they were all observational studies and, as their authors state, their design did not allow to establish any causality relationship. None of the four above-cited works evaluated the influence of instrumental birth as a potential risk factor for neonatal morbidity. Especially interesting is the study published by Zippori et al. [27]. This study was based on two large cohorts separated in time, with changes in the clinical management of the duration of the second stage of labour. In this study, it was found that the extension of the second stage of labour decreased the risk of a caesarean section but increased neonatal morbidity. However, the authors performed a confusion control through multivariate analysis techniques for the study of neonatal morbidity, only regarding maternal morbidity. In addition, changes may have occurred in the clinical practice regarding the second cohort with respect to the first one, which may have been responsible for the increase of neonatal morbidity.

Conversely, fewer recently published works have found similar results to those reported herein [7,10,11,12,13]. In this respect, it is worth highlighting the work by Gimovsky et al. [13], as it is the only randomised controlled trial conducted on this issue. These authors studied 78 nulliparous women and concluded that extending the duration of the second stage of labour beyond 1 h, as compared to the recommendations stated in traditional guidelines [8], was not related with increased neonatal morbidity. However, a potential limitation of this study was the relatively small number of women included. Besides, the trial was underpowered to detect small, but clinically important differences regarding the frequency of adverse outcomes between groups. The study by Altman et al. [11] also stands out as a systematic review that found no association between the duration of the second stage of labour and adverse neonatal outcomes. However, inherent methodological limitations were made evident in the studies. Recurrent limitations included the oversimplified categorisation of the second stage, inconsistencies between the study population characteristics, and the lack of control of confounding factors [11].

We consider that some of the differences observed between the aforementioned studies and the present study could be explained by the differences in the studied women, the different neonatal morbidity criteria used, and variability in the clinical practice of both centres and of the professionals involved (midwives, gynaecologists, anaesthesiologists, and paediatricians). Hence, caution is needed when making recommendations for setting a time limit of the second stage of labour if no maternal/foetal risk criteria exist. Setting a time limit of the second stage of labour as a single finishing criterion could increase the practice of instrumental birth and caesarean section, which would, in turn, increase the risk of other associated complications [8,22].

We observed how other factors such as the type of birth, maternal age, and neonatal birth weight are related to a high composite morbidity risk. Indeed, instrumental births had more than 2-fold risk of neonatal morbidity than normal births for nulliparous women, and this risk was more than 5-fold for multiparous women. In line with this, other authors have found similar findings [22,28,29]. However, it is necessary to be cautious about this association as our study design does not allow causality relationships. It was not possible to establish a separation between the morbidity produced exclusively by practicing instrumental birth and the morbidity before instrumental birth being practiced to lower it [30]. All in all, our results lead to considering restricting instrumental births in those clinical risk situations that may require this practice, and not just following the criterion of limiting the duration of the second stage of labour.

As for maternal age, multiparous women aged more than 35 years were at higher risk of composite neonatal morbidity. Our results were similar to those reported in other studies [31,32] in which advanced maternal age obtained worse neonatal outcomes. This could be due to the fact that signs of accelerated placental ageing, altered nutrient transport, and vascular function are observed in women of advanced maternal age [33].

Regarding the neonatal birth weight, an increased composite morbidity in those neonates whose birth weight was below 2500 g and who were born to multiparous women was found in our study. These results coincide with other studies done on this same issue [34,35].

The present study is not without its limitations, which are inherent to retrospective studies. For example, no records were taken of active maternal effort times. It would be valuable to know if maternal pushing time is related to neonatal morbidity, as well as to know other neonatal morbidity variables like being admitted to an NICU, long hospital stays for neonates, or possible complications that could appear in the long term. Other important limitations in our study are the lack of information on certain variables such as the presence of gestational diabetes, maternal body mass index, and the use of labour augmentation with oxytocin. Finally, another limitation was the lack of record of some umbilical cord arterial pH values, favoured by practicing delayed umbilical cord clamping and by determination errors. However, we considered that in most cases in which it was not possible to establish umbilical cord arterial pH values, the results would not be pathologic because our centre prioritises obtaining a sample before practicing delayed umbilical cord clamping if loss of foetal wellbeing may be expected.

On the other hand, this study has its strengths. It is the most recent study which finds no relationship between the duration of the second stage of labour and neonatal morbidity. We also employed techniques to control confounders by a multivariate analysis technique. Another strength is having employed objective variables like umbilical cord arterial pH and a variable that combines different criteria to obtain a more global neonatal morbidity assessment. 

## 5. Conclusions

The duration of the second stage of labour is not related with neonatal morbidity in our study population. Shortening this stage of labour with obstetric interventions should be based on criteria other than preventing neonatal adverse outcomes. This conclusion will allow for the reinforcement of the recommendation that the duration of the second stage of labour must not be exclusively limited by time criteria by conferring women more probabilities of vaginal birth.

## Figures and Tables

**Table 1:** Univariate analysis for neonatal morbidity in nulliparous women.

KERRYPNX	pH (*n* = 1554) (Missing = 164)		5-min Apgar Score (*n* = 1718)		Advanced Neonatal Resuscitation (*n* = 1718)		Composite Neonatal Morbidity (*n* = 1718)	
**Variables**	**<7.10**	**≥7.10**	**<7**	**≥7**	**Yes**	**No**	**Yes**	**No**
**Neonatal birth weight (g)**								
2500–3999 g	31 (2.1)	1412 (97.9)	5 (0.3)	1593 (99.7)	54 (3.4)	1544 (96.6)	81 (5.1)	1517 (94.9)
<2500 g	1 (1.4)	69 (98.6)	1 (1.3)	76 (98.7)	3 (3.9)	74 (96.1)	3 (3.9)	74 (96.1)
>4000 g	1 (2.4)	40 (97.6)	0 (0.0)	43 (100.0)	0 (0.0)	43 (100.0)	1 (2.3)	42 (97.7)
***p* Value**	0.91		0.33		0.45		0.65	
**Labour Induction**								
No	23 (2.1)	1087 (97.9)	4 (0.3)	1230 (99.7)	34 (2.8)	1200 (97.2)	54 (4.4)	1180 (95.6)
Yes	10 (2.3)	434 (97.9)	2 (0.4)	482 (99.6)	23 (4.8)	461 (95.2)	31 (6.4)	453 (93.6)
***p* Value**	0.82		0.78		**0.04**		0.08	
**Gestational Age (weeks)**								
<37	1 (1.7)	58 (98.3)	1 (1.6)	62 (98.4)	3 (4.8)	60 (95.2)	3 (4.8)	60 (95.2)
37–41	25 (1.9)	1283 (98.2)	4 (0.3)	1445 (99.7)	45 (3.1)	1404 (96.9)	67 (4.6)	1382 (95.4)
>41	7 (3.7)	180 (96.3)	1 (0.5)	205 (99.5)	9 (4.4)	197 (95.6)	15 (7.3)	191 (92.7)
***p* Value**	0.26		0.21		0.51		0.26	
**Duration First Stage of Labour**								
Until 3 h	7 (1.7)	402 (98.3)	1 (0.2)	454 (99.8)	10 (2.2)	445 (97.8)	15 (3.3)	440 (96.7)
3–6 h	14 (2.2)	614 (97.8)	2 (0.3)	704 (99.7)	14 (2.0)	692 (98.0)	27 (3.8)	679 (96.2)
6–9 h	8 (2.3)	338 (97.7)	2 (0.5)	364 (99.5)	19 (5.2)	347 (94.8)	26 (7.1)	340 (92.9)
>9 h	4 (2.3)	167 (97.7)	1 (0.5)	190 (99.5)	14 (7.3)	177 (92.7)	17 (8.9)	174 (91.1)
***p* Value**	0.93		0.83		**<0.001**		**0.002**	
**Duration Second Stage of Labour**								
Until 1 h	9 (1.7)	532 (8.3)	2 (0.3)	601 (99.7)	15 (2.5)	588 (97.5)	24 (4.0)	579 (96.0)
1–2 h	12 (2.8)	419 (97.2)	2 (0.4)	469 (99.6)	19 (4.0)	452 (96.0)	27 (5.7)	444 (94.3)
2–3 h	7 (2.3)	294 (97.7)	1 (0.3)	339 (99.7)	12 (3.5)	328 (96.5)	19 (5.6)	321 (94.4)
≥3 h	5 (1.8)	276 (98.2)	1 (0.3)	303 (99.7)	11 (3.6)	293 (96.4)	15 (4.9)	289 (95.1)
***p* Value**	0.64		0.99		0.54		0.55	
**Epidural Analgesia**								
No	2 (1.9)	106 (98.1)	0 (0.0)	121 (100.0)	1 (0.8)	120 (99.2)	3 (2.5)	118 (97.5)
Yes	31 (2.1)	1415 (97.9)	6 (0.4)	1591 (99.6)	56 (3.5)	1541 (96.5)	82 (5.1)	1515 (94.9)
***p* Value**	*0.84*		*0.50*		*0.11*		*0.19*	
Mode of birth								
Normal birth	22 (1.7)	1298 (98.3)	4 (0.3)	1463 (99.7)	41 (2.8)	1426 (97.2)	60 (4.1)	1407 (95.9)
**Instrumental birth**	11 (4.7)	223 (95.3)	2 (0.8)	249 (99.2)	16 (6.4)	235 (93.6)	25 (10.0)	226 (90.0)
***p* Value**	**0.003**		0.19		**0.003**		**<0.001**	
**Maternal age (years)**								
≤35	26 (1.9)	1342 (98.1)	5 (0.3)	1510 (99.7)	47 (3.1)	1468 (96.9)	70 (4.6)	1445 (95.4)
>35	7 (3.8)	179 (96.2)	1 (0.5)	202 (99.5)	10 (4.9)	193 (95.1)	15 (7.4)	188 (92.6)
***p* Value**	0.09		0.71		0.17		0.09	

Bold: Significant results are highlighted.

**Table 2:** Univariate analysis for neonatal morbidity in multiparous women.

	pH (n = 1912) (Missing = 233)		5-min Apgar Score (n = 2145)		Advanced Neonatal Resuscitation (n = 2145)		Composite Neonatal Morbidity (n = 2145)	
**Variables**	**<7.10**	**≥7.10**	**<7**	**≥7**	**Yes**	**No**	**Yes**	**No**
**Neonatal Birth Weight (g)**								
2500–3999 g	37 (2.1)	1694 (97.9)	3 (0.2)	1944 (99.8)	37 (1.9)	1910 (98.1)	68 (3.5)	1879 (96.5)
<2500 r	2 (3.4)	57 (96.6)	0 (0.0)	66 (100.0)	4 (6.1)	62 (93.9)	6 (9.1)	60 (90.9)
>4000 g	1 (0.8)	121 (99.2)	0 (0.0)	132 (100.0)	1 (0.8)	131 (99.2)	2 (1.5)	130 (98.5)
***p* Value**	0.48		0.86		**0.03**		**0.02**	
**Labour Induction**								
No	24 (1.6)	1475 (98.4)	2 (0.1)	1685 (99.9)	27 (1.6)	1660 (98.4)	47 (2.8)	1640 (97.2)
Yes	16 (3.9)	397 (96.1)	1 (0.2)	457 (99.8)	15 (3.3)	443 (96.7)	29 (6.3)	429 (93.7)
***p* Value**	**0.004**		0.61		**0.02**		**<0.001**	
**Gestational Age (weeks)**								
<37	1 (1.7)	59 (98.3)	0 (0.0)	66 (100.0)	1 (1.5)	65 (98.5)	2 (3.0)	64 (97.0)
37–41	36 (2.2)	1614 (97.8)	3 (0.2)	1850 (99.8)	34 (1.8)	1819 (98.2)	64 (3.5)	1789 (96.5)
>41	3 (1.5)	199 (98.5)	0 (0.0)	226 (100.0)	7 (3.1)	219 (96.9)	10 (4.4)	216 (95.6
***p* Value**	0.79		0.79		0.42		0.74	
**Duration First Stage of Labour**								
Until 3 h	17 (1.6)	1020 (98.4)	2 (0.2)	1176 (99.8)	13 (1.1)	1165 (98.9)	27 (2.3)	1151 (97.7)
3–6 h	13 (2.0)	622 (98.0)	1 (0.1)	710 (99.9)	17 (2.4)	694 (97.6)	29 (4.1)	682 (95.9)
6–9 h	7 (3.9)	173 (96.1)	0 (0.0)	191 (100.0)	7 (3.7)	184 (96.3)	13 (6.8)	178 (93.2)
>9 h	3 (5.0)	57 (95.0)	0 (0.0)	65 (100.0)	5 (7.7)	60 (92.3)	7 (10.8)	58 (89.2)
***p* Value**	0.09		0.93		**<0.001**		**<0.001**	
**Duration Second Stage of Labour**								
Until 1 h	27 (1.9)	1385 (98.1)	2 (0.1)	1587 (99.9)	22 (1.4)	1567 (98.6)	46 (2.9)	1543 (97.1)
1–2 h	7 (2.3)	291 (97.7)	0 (0.0)	327 (100.0)	13 (4.0)	314 (96.0)	18 (5.5)	309 (94.5)
2–3 h	3 (2.0)	145 (98.0)	1 (0.6)	164 (99.4)	4 (2.4)	161 (97.6)	6 (3.6)	159 (96.4)
≥3 h	3 (5.6)	51 (94.4)	0 (0.0)	64 (100.0)	3 (4.7)	61 (95.3)	6 (9.4)	58 (90.6)
***p* Value**	0.32		0.37		**0.006**		**0.007**	
**Epidural Analgesia**								
No	7 (1.7)	397 (98.3)	0 (0.0)	470 (100.0)	0 (0.0)	470 (100.0)	7 (1.5)	463 (98.5)
Yes	33 (2.2)	1475 (97.8)	3 (0.2)	1672 (99.8)	42 (2.5)	1633 (97.5)	69 (4.1)	1606 (95.9)
***p* Value**	0.57		0.36		**0.001**		**0.006**	
**Mode of Birth**								
Normal birth	33 (1.8)	1812 (98.2)	3 (0.1)	2067 (99.9)	33 (1.6)	2037 (98.4)	61 (2.9)	2009 (97.1)
Instrumental birth	7 (10.4)	60 (89.6)	0 (0.0)	75 (100.0)	9 (12.0)	66 (88.0)	15 (20.0)	60 (80.0)
***p* Value**	**<0.001**		0.74		**<0.001**		**0.013**	
**Maternal Age (years)**								
≤35	21 (1.6)	1324 (98.4)	1 (0.1)	1511 (99.9)	23 (1.5)	1489 (98.5)	41 (2.7)	1471 (97.3)
>35	19 (3.4)	548 (96.6)	2 (0.3)	631 (99.7)	19 (3.0)	614 (97.0)	35 (5.5)	598 (94.5)
***p* Value**	**0.01**		0.16		**0.02**		**0.001**	
**Previous Cesarean Birth**								
No	31 (1.8)	1683 (98.2)	2 (0.1)	1926 (99.9)	34 (1.8)	1894 (98.2)	60 (3.1)	1868 (96.9)
Yes	9 (4.5)	189 (95.5)	1 (0.5)	216 (99.5)	8 (3.7)	209 (96.3)	16 (7.4)	201 (92.6)
***p* Value**	**0.011**		0.18		0.05		**0.001**	

Bold: Significant results are highlighted.

**Table 3:** Multivariate analysis for composite neonatal morbidity in nulliparous and multiparous women.

	Nulliparous Women (*n* = 1718)		Multiparous Women (*n* = 2145)	
Variables	OR (95% CI)	*p* Value	OR (95% CI)	*p* Value
**Neonatal Birth Weight (g)**				
2500–3999 g (Reference)		0.78		0.07
<2500 g	0.83 (0.24–2.87)	0.78	2.78 (1.02–7.58)	**0.04**
>4000 g	0.42 (0.06–3.17)	0.40	0.47 (0.11–1.98)	0.30
**Labour Induction**		0.65		0.13
No (Reference)				
Yes	1.12 (0.69–1.84)		1.52 (0.88–2.62)	
**Gestational Age (weeks)**		0.61		0.55
37–41 (Reference)				
<37	1.14 (0.33–3.90)	0.84	0.47 (0.10–2.11)	0.32
>41	1.36 (0.74–2.49)	0.32	1.17 (0.57–2.40)	0.68
**Duration First Stage of Labour**		0.08		0.30
Until 3 h (Reference)				
3–6 h	0.99 (0.51–1.95)	0.98	1.39 (0.79–2.46)	0.25
6–9 h	1.70 (0.84–3.44)	0.14	1.62 (0.75–3.50)	0.22
>9 h	2.06 (0.94–4.50)	0.07	2.37 (0.90–6.25)	0.08
**Duration Second Stage of Labour**		0.59		0.48
Until 1 h (Reference)				
1–2 h	1.30 (0.73–2.33)	0.37	1.39 (0.76–2.51)	0.28
2–3 h	1.10 (0.58–2.11)	0.76	0.87 (0.35–2.14)	0.76
≥3 h	0.83 (0.41–1.68)	0.60	1.79 (0.66–4.85)	0.25
**Epidural Analgesia**		0.58		0.20
No (Reference)				
Yes	1.42 (0.41–4.96)		1.73 (0.74–4.04)	
**Type of Birth**		**0.002**		**<0.001**
Normal birth (Reference)				
Instrumental birth	2.33 (1.38–3.96)		5.37 (2.73–10.56)	
Maternal Age (years)		0.23		**0.006**
≤35 (Reference)				
>35	1.44 (0.79–2.60)		1.97 (1.22–3.17)	

Bold: Significant results are highlighted.
